# A Global Landscape of Miniature Inverted-Repeat Transposable Elements in the Carrot Genome

**DOI:** 10.3390/genes12060859

**Published:** 2021-06-03

**Authors:** Alicja Macko-Podgórni, Gabriela Machaj, Dariusz Grzebelus

**Affiliations:** Department of Plant Biology and Biotechnology, University of Agriculture in Krakow, 31-120 Kraków, Poland; g.machaj@student.urk.edu.pl (G.M.); d.grzebelus@urk.edu.pl (D.G.)

**Keywords:** *Daucus carota*, genome annotation, MITE, alternative splicing, gene expression

## Abstract

Miniature inverted-repeat transposable elements (MITEs) are the most abundant group of Class II mobile elements in plant genomes. Their presence in genic regions may alter gene structure and expression, providing a new source of functional diversity. Owing to their small size and lack of coding capacity, the identification of MITEs has been demanding. However, the increasing availability of reference genomes and bioinformatic tools provides better means for the genome-wide identification and analysis of MITEs and for the elucidation of their contribution to the evolution of plant genomes. We mined MITEs in the carrot reference genome DH1 using MITE-hunter and developed a curated carrot MITE repository comprising 428 families. Of the 31,025 MITE copies spanning 10.34 Mbp of the carrot genome, 54% were positioned in genic regions. *Stowaways* and *Tourists* were frequently present in the vicinity of genes, while *Mutator*-like MITEs were relatively more enriched in introns. *hAT*-like MITEs were relatively more frequently associated with transcribed regions, including untranslated regions (UTRs). Some carrot MITE families were shared with other Apiaceae species. We showed that *hAT*-like MITEs were involved in the formation of new splice variants of insertion-harboring genes. Thus, carrot MITEs contributed to the accretion of new diversity by altering transcripts and possibly affecting the regulation of many genes.

## 1. Introduction

Miniature inverted-repeat transposable elements (MITEs) are non-autonomous Class II mobile elements, widespread and abundant in plant genomes. MITEs constitute an artificial group within class II elements characterized by high copy numbers, small size (>700 bp) and lack of any coding capacity [1]. They are further divided into sub-groups based on the similarity of their terminal inverted repeats (TIRs) and target site duplications (TSDs) to those of certain elements representing major superfamilies of Class II transposons. *Tourists* and *Stowaways* were the first described and the most thoroughly characterized MITE sub-groups derived from *PIF/Harbinger* and *Tc1/Mariner* elements, respectively [2,3]. Subsequently, MITEs related to other DNA transposons, i.e., *Mutators* and *hATs*, were also reported [4,5]. The similarity of MITE TIRs to those of autonomous DNA transposons allows MITEs to be mobilized by transposases encoded by their autonomous relatives, resulting in the transposition and formation of characteristic TSDs upon insertion.

MITEs are often inserted in the vicinity of genes. Such a location entails the possibility of a functional impact on the nearby gene. Besides the knockout mutations resulting from a disruption of the gene structure [6,7,8], numerous reports have been published indicating the possibility that MITE insertions may alter the expression of adjacent genes [9,10,11,12,13,14]. MITEs can also give rise to siRNAs, and drive gene silencing through RNA-directed methylation (RdDM) [14,15]. It has been shown that genes containing MITE insertions in their promoters may be epigenetically down-regulated, e.g., MITE-associated methylation of *ZmCCT* and *ZmNAC111* promoters in maize resulted in an early-flowering phenotype and a drought-sensitive phenotype of maize seedlings, respectively [16,17]. MITEs may also carry transcription factor binding sites (TFBS) [18,19] or cis-acting DNA regulatory elements, e.g., new splice sites [20], transcription start sites, TATA boxes, and polyadenylation signals [21]. Insertions of MITEs into 3’ UTRs may considerably affect post-transcriptional gene regulation, through modulation of RNA stability or translation [21,22,23], while insertion into the upstream region of a gene may result in its up-regulation [24,25]. However, in order to investigate the impact of MITEs on host genes, their genome-wide identification and characterization is an essential prerequisite.

Plant genomes differ in terms of MITE content and diversity. The number of MITE copies can vary sharply even among closely related species. For example, the number of MITE copies in the genome of *Arabidopsis lyrata* is more than five times higher than in *Arabidopsis thaliana*, while the genome of the former is only twice as large as that of the latter [26]. Similarly, watermelon, having a slightly smaller genome than melon, contains seven times more MITE copies [26]. High levels of MITE diversity have been observed in Poaceae, while within dicot plants, species characterized by high MITE diversity are less common [26]. For example, in the grapevine genome, only eight MITE families were identified, and neither *Stowaway* MITEs nor related autonomous *Tc1/Mariners* were found [27,28]. It is not clear what mechanism is responsible for such diversification among plants.

The carrot (*Daucus carota* subsp. *sativus* Hoffm.) is an economically important crop, and a significant source of β-carotene in the human diet [29]. Although the carrot has a relatively small genome of ca. 473 Mb, the repetitive fraction constitutes around 46%, of which 13.6% is attributed to Class II DNA transposons [30]. To date, two groups of carrot MITEs have been more thoroughly characterized, i.e., *Krak* elements belonging to the *Tourist* group [31] and *DcSto* elements attributed to the *Stowaway* group [32,33]. Owing to their high insertional polymorphism, they were utilized as sources of molecular markers [34,35,36]. It was shown that *DcSto* elements were associated with genes, most frequently occurring in 5′ and 3′ UTRs. A significant enrichment of gene encoding transcription factors was also revealed in the fraction of genes associated with *DcSto* insertions in their 2 kb up- and downstream regions, suggesting the possible functional impact of those MITEs [33]. Nevertheless, a comprehensive, global annotation of carrot MITEs has been lacking.

Here, we report on the global characterization of carrot MITEs, their genomic distribution, association with genes and presence in transcripts. We provided examples of genic insertions of *hAT*-like MITEs resulting in the formation of novel splice variants. We also identified MITEs in genomes of other Asterid species and showed that the carrot, despite its relatively small genome, was characterized by an exceptional diversity and abundance of MITEs. The curated carrot MITE repository will facilitate research on the origin of carrot MITEs and their role in the host genome.

## 2. Materials and Methods

### 2.1. Identification and Genomic Localization of MITEs in the Carrot Reference Genome

Carrot MITE families were identified with MITE-Hunter [37], using default parameters. The final dataset, comprising 522 MITE consensus sequences, was manually curated and grouped into 428 MITE families using the 80-80-80 similarity rule [1]. Those sequences were used as queries for a blastn search [38] against the carrot DH1 reference genome (GenBank assembly accession number: GCA_001625215.1; [30]). The blastn output was parsed using a custom Perl script, with parameters allowing the extraction of coordinates of copies following 80–80–80 similarity rule, and for which similarity at both ends started between 1–10 nucleotides and continued over the whole query sequence. Genomic regions meeting those criteria were used to produce two bed files, one with exact coordinates and the other with coordinates extended by 50 nt at both ends. The bed file with the MITE-flanking regions was used to extract sequences that were manually inspected to verify/identify MITE TIRs and TSDs. Subsequently, TSDs were used to combine families into groups related to DNA transposons [Class II], i.e., *Stowaways* (related to *Tc1/mariner*), *Tourists* (related to *PIF/Harbinger*), *hAT*-like, and *Mutator*-like MITEs.

To mine for autonomous elements related to MiMs (MITEs inserted in microsatellite) [26], we used blastx implemented in blastall (v 1 -b 1) to search for *Mutator* transposases in the carrot reference genome. As queries, we used *Mutator* proteins identified in *Oryza sativa* (AAX92869.1), *Rosa chinensis* (PRQ51974.1), *Helianthus annuus* (OTF91787.1), *Solanum tuberosum* (ABI34394.1), *Arabidopsis thaliana* (AAG51216.1), and *Medicago truncatula* [39]. Genomic regions with similarity to the *Mutator* proteins were extracted with 4 kb flanking sequences and used to identify TIRs. TIRs were recognized based on the results of the self-blast of extracted sequences (blastn; -v 1 -b 1), from which the coordinates of hits present on both flanks of the putative transposase coding region were in complementary orientation and shared at least 50% identity. They were used to define the boundaries of putative autonomous elements. Subsequently, we used blastn with default parameters to compare 50-bp-long ends of consensus MiM sequences with the mined *Mutator*-like elements.

A bed file containing coordinates of MITEs was used to determine the positions of MITE copies in the context of genic regions, as described by Macko-Podgórni et al. [33]. MITE copies were categorized based on the NCBI carrot genome annotation (GCF_001625215.1_ASM162521v1_genomic.gff) as 5′UTR (MITE coordinates overlapping annotated 5′UTRs), 3′UTR (MITE coordinates overlapping annotated 3′UTRs), cds (MITE coordinates overlapping annotated cds), intron (MITE coordinates located within an annotated intron), upstream (MITE present within 1 kb upstream a gene), downstream (MITE present within 1 kb downstream a gene), while copies not fulfilling any of those criteria were considered as intergenic. All calculations were performed using the bed file in R [40]. A gff3 file showing the annotation of carrot MITEs in the reference genome was developed (File S1 in the Supplementary Materials).

### 2.2. Carrot MITEs in Transcribed Regions

RNAseq reads from twenty DH1 tissues (PRJNA291977; Supplementary Table S2, [30]) were used to identify MITE-containing transcripts. Reads mapping to MITE sequences were found using Bowtie2 v. 2.3.5.1 [41], extracted with SAMtools v. 1.9 [42], blasted against the carrot DH1 reference genome with blastall [38] with the following parameters: -p blastn -F F -v 1 -b 1 -K 1. Blast hits longer than 20 bp with a similarity higher than 80% were kept. Subsequently, regions with blast hits were overlapped with MITE annotation using BedTools v. 2.26.0 [43]. If at least ten reads mapped to the MITE sequence, the corresponding gene with a MITE insertion (upstream, downstream, in 5’UTR, 3’UTR, cds, or intron) was further analyzed.

To determine the expression of MITE-containing and non-MITE-containing isoforms, RNAseq reads from DH1 were mapped to the carrot DH1 reference genome (GenBank assembly accession number: GCA_001625215.1 [30], Table S1 in the Supplementary Materials) using STAR v. 2.7.3a [44] with the following parameters: outSAMmapqUnique: 50; outFilterMultimapNmax: 20; alignSJoverhangMin: 8; alignSJDBoverhangMin: 1; outFilterMismatchNmax: 999; outFilterMismatchNoverLmax: 0.04; alignIntronMin: 20; alignIntronMax: 1,000,000; and alignMatesGapMax: 1,000,000. Novel isoform identification and calculation were performed according to Machaj et al. [45]. Subsequently, SAMtools v. 1.9 [42] and BedTools v. 2.26.0 [43] were used to extract MITE and gene coverage from bam files. MITE/gene (M/g) ratios were calculated based on normalized RPK (reads per kilobase; RPK/(library size/1000000)) values and genes with RPK > 1 and the M/g ratio > 1.9 were further evaluated. Analysis of the differential exon usage, resulting from the presence of a MITE copy, was performed using JunctionSeq [46] based on RNAseq data from seven carrot tissues differing with respect to the number of normalized RNAseq reads attributed to MITEs. The differential exon usage was tested between “bracts (from not opened flower), from 2 cm umbel” (SRR2148980) as a reference, and each of the six tissues: “callus” (SRR2148992), “fibrous roots” (SRR2148991), “whole flowers (not opened), 2 cm umbel” (SRR2148981), “leaves stage 1, 0.5–1 cm young sprout” (SRR2148984), “stressed root, whole storage root” (SRR2148997), and “germinating seeds, at the beginning of germination” (SRR2148999). In total, 40 genes carrying copies of *hAT*-like MITEs were investigated. Expression levels of those genes were represented as a heatmap, created in R [40].

### 2.3. Mining for MITEs in the Reference Genomes of Other Asterid Species

The mining strategy used to identify carrot MITEs was applied to eight reference genomes representing members of the Asterid clade (Supplementary Table S2 [47,48,49,50,51,52,53]). Then, blastn [38] was used for among-species comparisons of MITE consensus sequences to identify MITE families shared among Asterids. The presence of a region covering a minimum of 60% MITE length with identity equal to or higher than 60% was used as a threshold to define related MITE families from different genomes. A graphical representation of levels of similarity among related MITEs was drawn using Circoletto [54], with the following blastn parameters: -F F -e 1e-10 -E -1 -v 200 -b 200, and “score/max” ratio coloring with blue ≤ 0.25, green ≤ 0.50, orange ≤ 0.75, red > 0.75.

## 3. Results

### 3.1. Abundance and Genomic Localization of Carrot MITEs

**Figure 1:**
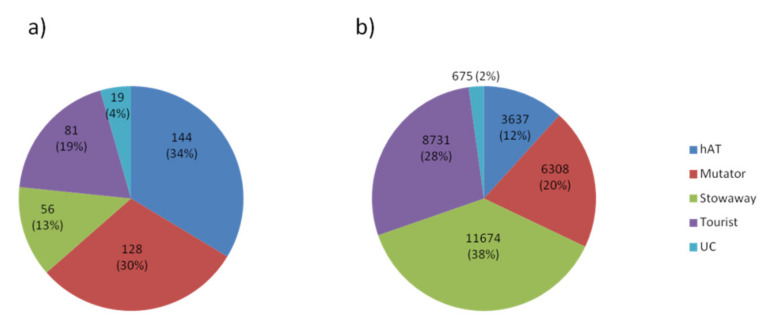
Abundance of MITE groups in the carrot genome with respect to the number of families (**a**) and the number of copies (**b**). UC stands for unclassified MITEs.

We identified 428 MITE families in the carrot reference genome and divided them into groups on the basis of their relationships to Class II TE superfamilies, as revealed by their TIR and TSD similarity (Figure 1). We used consensus sequences representing each family to identify individual copies along the reference assembly of the carrot genome [30]. In total, MITEs were estimated to occupy around 2% of the carrot genome (31,025 copies spanning 10.34 Mbp).

The largest genome fraction was attributed to *Stowaways* (2.97 Mbp) followed by *Tourists* (2.78 Mbp) and *Mutator*-like MITEs (2.71 Mbp). *hAT*-like MITEs were the least numerous but the most diverse, comprising one-third of all identified MITE families. Families derived from *Mutator*-like elements accounted for ca. 30%, while *Tourists* and *Stowaways* grouped 19% and 13% of MITE families, respectively (Table 1). More than half of all MITEs (16,693 copies; 54%) were inserted within genic regions, defined as 1 kb upstream and downstream of genes and including the gene body (Table 1). We also identified 2156 copies belonging to 15 families flanked by “TA” stretches and having distinguishable but usually poorly conserved TIRs. For three of those families, we were able to identify related putative autonomous *Mutator* elements, confirming that they should be attributed to the group of *Mutator*-like MITEs, and classified them as MiMs, following the nomenclature of Chen et al. [26].

**Figure 2:**
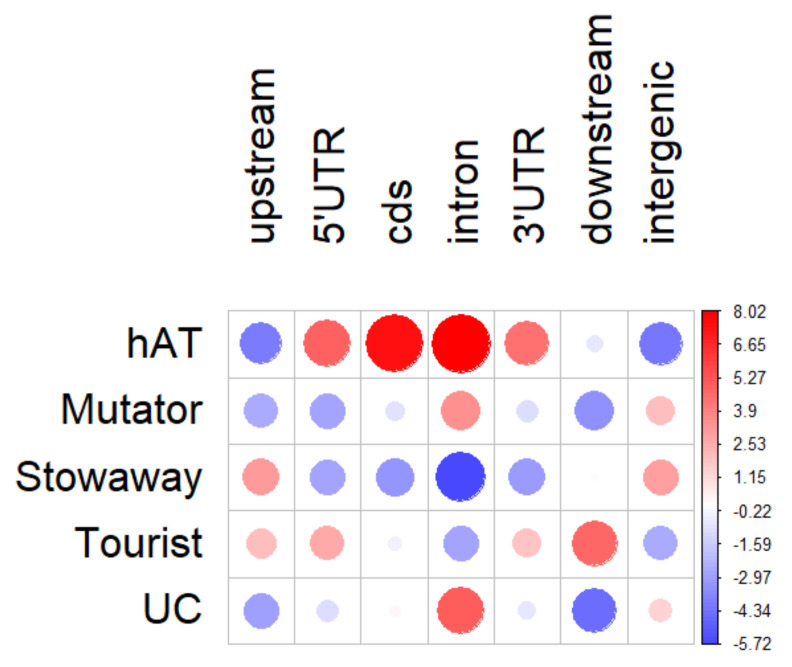
Relative abundance of MITE groups in different genomic regions (*p*-value = 0.0005). The color scale reflects deviations from average values. Circle size is proportional to the contribution of each test to the total Pearson chi-squared score. UC stands for unclassified MITEs.

The MITE groups differed in terms of their copy number, the average length of the element and genomic localization. *Stowaways* were on average 254 bp-long, and constituted the most abundant group of MITEs in the carrot genome, reaching 11,674 copies. The largest family, comprising 1452 copies, was attributed to that group. The most numerous *Stowaway* families included previously described *DcSto* families [33]. *Stowaway* elements were relatively enriched in intergenic regions and upstream from genes. *Tourists* were represented by 8731 copies with an average element length of 319 bp. The largest family in that group comprised 1353 copies. A previously described *Krak* family [31], comprising 333 copies in the carrot reference genome, was the seventh-largest family of *Tourists*. *Tourists* were more frequently positioned upstream and downstream from genes and in UTRs. *Mutator*-like MITEs comprised 6308 copies, with a mean length of 429 bp. The largest family within that group contained 542 elements. *Mutator*-like MITEs, including MiMs, were present mainly in intergenic regions and in introns. The group of *hAT*-like elements was the least numerous, represented by 3637 copies with an average length of 454 bp and the largest family comprising 217 copies. *hAT*-like elements were relatively more frequently located in coding regions and UTRs, as compared to the other MITE groups (Table 1, Figure 2, Figure S1, File S2 in the Supplementary Materials).

### 3.2. Carrot MITEs Are Co-Transcribed with Genes and Are Involved in Tissue-Specific Alternative Splicing

In order to determine whether MITEs localized in the vicinity of genes were co-transcribed, we used RNAseq reads from 20 carrot tissues [30] and mapped them onto MITE sequences to search for MITEs covered by more than ten reads. In total, 3469 MITE copies, representing 336 families, were retrieved. Of those, 60% were localized in introns, 16% and 14% 1 Kb upstream or downstream from genes, respectively, 4% in 5’UTR, 5% in 3’UTR, and 1% in cds (Table S3, Figure S2 in the Supplementary Materials). The number of copies belonging to particular MITE families, residing in transcribed regions, correlated with the total number of copies in those families (*p*-value = 2.2 × 10^−16^ (Supplementary Figure S3)). *hAT*-like elements, which were shown to be relatively more frequent in gene bodies, were also more frequently co-transcribed. Interestingly, *hAT*-like elements located in introns were frequently retained in transcripts, while intronic *Stowaways* and *Mutator*-like MITEs were usually spliced out together with introns and did not significantly contribute to transcripts (Figure S4 in the Supplementary Materials). Thus, we were prompted to investigate if *hAT*-like MITEs might be involved in the formation of novel gene-splicing variants.

In some tissues, we observed more reads mapping to MITEs, possibly indicating a higher expression of MITE-containing isoforms. The highest normalized RPK values were shown for “stressed leaves of 7–8 cm at reversible wilting point”, “stressed leaves of 2–2.5 cm at reversible wilting point” and “fibrous roots” (Figure S5 in the Supplementary Materials). Because such differences might have reflected general differences in gene expression, not related to the presence of MITEs, we calculated the MITE vs. gene (M/g) expression ratio, in which a normalized number of reads mapping to the co-transcribed MITE segment was divided by a normalized number of reads mapping to the whole gene, providing means to determine if the MITE-derived isoform was indeed differentially expressed in a tissue-specific manner. In total, we identified 3022 genes with normalized RPK > 1 and M/g > 1.9. Of those, 63%, 26% and 11% of genes carried MITEs localized in introns, upstream or downstream regions of genes, and transcripts, respectively (Table S4, File S2 in the Supplementary Materials).

**Figure 3:**
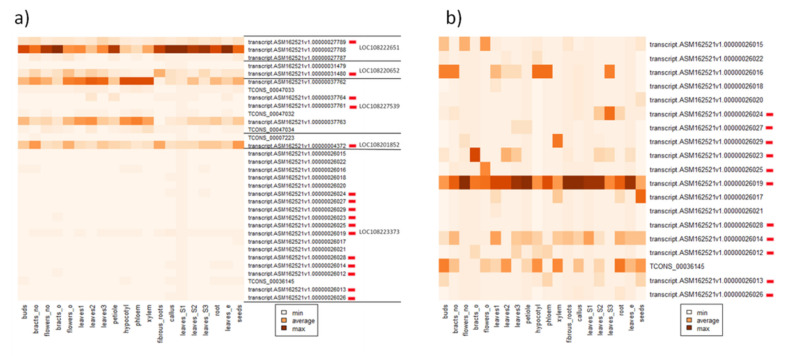
Heatmap of the expression (TPM, transcript per million) of five genes (**a**) and LOC108223373 (**b**) producing *hAT*-containing isoforms (labeled by red rectangles). Expression was normalized by tissue type to allow comparisons between genes and isoforms. Novel isoforms are labeled as TCONS_XXX. Detailed results/descriptions are provided in Supplementary File S3.

To test the possible impact of MITEs on the differential usage of exons in different tissues, we retrieved genes for which differences in the maximum and minimum M/g values were higher than 1.5. From those, we randomly selected 40 genes carrying *hAT*-like elements in their UTRs, cds or introns (Table S5 in the Supplementary Materials). We found five genes for which the differential exon usage was reported, for isoforms containing *hAT*-like MITEs (Figure S6–S10; File S3 in the Supplementary Materials). In the case of two genes (LOC108223373 and LOC108201852), the presence of *hAT*-like MITEs led to the formation of isoforms encompassing the whole MITE. The *hAT*-like MITE present in LOC108227539 was partially incorporated into the new exon, while MITEs within the other two genes (LOC108220652 and LOC108222651) were co-transcribed as alternative 5’UTR and 3’UTR, respectively. In general, regardless of the tissue, we observed the prevalence of one isoform. In the case of LOC108222651 and LOC108227539, the non-MITE-containing variant was generally more abundant; however, the expression of MITE-containing isoforms differed slightly among tissues. The expression of LOC108223373 was lower than the other genes (Figure 3a; File S3, in the Supplementary Materials), mostly the MITE-containing isoform was produced, and its expression levels differed depending on the tissue (Figure 3b; File S3 in the Supplementary Materials). For the remaining two genes (LOC108201852 and LOC108220652), MITE-containing isoforms were predominant. The non-MITE-containing isoform of LOC108201852 was expressed only in “bracts (from not opened flower)”, while the MITE-containing isoform was highly expressed in bracts (not open), open flowers, fibrous roots and seeds. The MITE-containing isoform of the LOC108220652 gene was expressed in all tissues, and the highest expression was reported for bracts (not open), open flowers, leaves, hypocotyls, phloem, and xylem. The non-MITE-containing isoform was observed in the callus and leaves.

### 3.3. Identification of MITEs in Asterids

**Figure 4:**
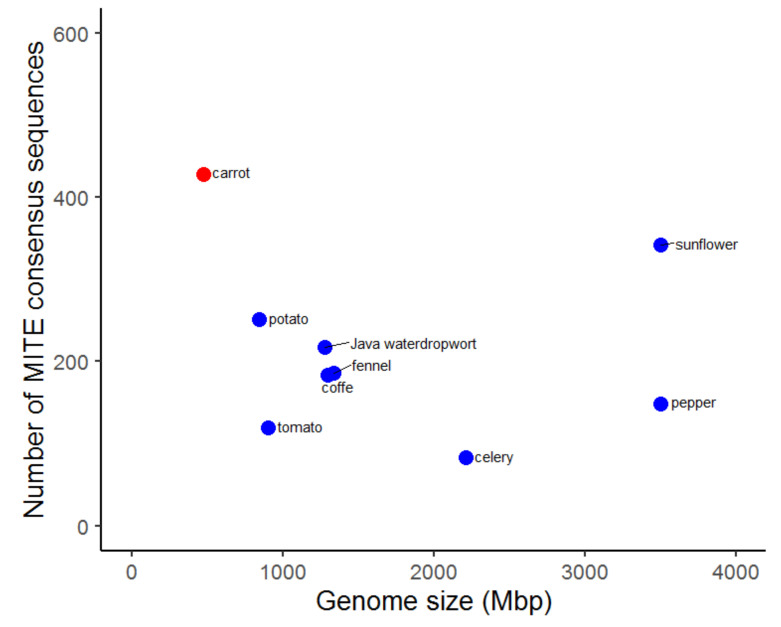
Plot showing the number of MITE families found in Asterid species (*y* axis) related to the genome size (*x* axis).

In order to compare the abundance of MITEs in the carrot genome to those in other Asterid species, we mined MITEs from eight Asterid reference genomes. The number of MITE families and the genome fraction occupied by MITEs were not correlated with the genome size (*p*-values = 0.37 and 0.79, respectively). The highest abundance and diversity of MITEs (428 families) was observed in the carrot genome, while only 83 MITE families were identified in the celery genome (Figure 4; Table 2).

In addition, the largest proportion of the genome was attributed to MITE elements in the carrot (2.19%, 10.34 Mbp), followed by the sunflower (2.09%, 73.28 Mbp), celery (1.25%, 27.71 Mbp), coffee (0.93%, 12.11 Mbp), potato (0.91%, 7.65 Mbp), and tomato (0.79%, 7.07 Mbp). MITEs were the least abundant in fennel (0.42%, 5.67 Mbp) and pepper (0.36%, 12.43 Mbp). In some investigated genomes, e.g., celery, high MITE copy numbers were observed despite a limited diversity at the family level (Figure S11 in the Supplementary Materials).

MITEs present in genomes of Asterids from different families (Apiaceae, Solanaceae, Rubiaceae, Asteraceae) revealed no similarity above the threshold of 60% sequence identity over 60% of the sequence length. More similar MITEs were identified within Solanaceae (tomato, potato and pepper) and within Apiaceae (carrot, fennel, celery, and Java water dropwort) (Table S6 in the Supplementary Materials). Thus, the number of related MITEs in different genomes corresponded to phylogenetic relationships among the species. In total, similar counterparts in Apiaceae were found for 150 carrot MITE families (Table S7, File S4 in the Supplementary Materials). Of those, nine MITE families were present in the four Apiaceae species, with six families representing *Stowaways* (*DcSto1*, *DcSto4*, *DcSto6*, *DcSto10*, *DcSto29*, and *DcSto37*), two families representing *Tourists,* and one *hAT*-like MITE family (Figure S12 in the Supplementary Materials). Forty-three carrot MITE families were also present in two other Apiaceae species (20 shared with Java water dropwort and fennel, 18 with celery and fennel, and five with Java water dropwort and celery). The remaining 95 carrot MITE families were similar to MITEs from one other Apiaceae species (53, 27 and 15 were shared with fennel, Java water dropwort and celery, respectively).

## 4. Discussion

### 4.1. Diversity and Abundance of Carrot MITEs

MITEs are the most abundant group of DNA transposons in plant genomes. Higher diversity and abundance of MITEs have also been reported in monocots than in dicots [26]. We identified 428 families comprising 31,025 copies in the carrot genome. Thus, the carrot stands out from most other dicot species and can be placed among few species, having relatively small genomes but containing large numbers of diverse MITEs, along with *Medicago truncatula* and mulberry as the most prominent examples. In the *M. truncatula* genome of 307 Mbp, 288 MITE families and 132,834 copies were identified [26] while the 357 Mb-long mulberry genome was reported to contain 90,789 full-length copies divided into 232 families [20]. Thus, even though the MITE copy number in the carrot is lower, they show remarkable diversity.

We identified 15 families, grouping more than 2000 MiMs residing in “TA” stretches. MITEs preferably inserted into microsatellites have been described in other plant genomes. Chen et al. [26] reported that MiMs were present in ten of 41 plant species [26]. MiMs were first described in rice as high copy-number MITEs having poorly recognizable TIRs and preferentially inserting into “TA” repeats [55]. MiMs from other species were characterized by similar features and, based on the similarity of their TIRs to related autonomous elements, they were attributed to the *Mutator* superfamily [39,56]. It has been suggested that the propensity to insert into “TA” stretches might reflect a strategy adopted to avoid removal from the genome by positioning into regions not subject to strong selective pressure [39]. However, it remains unclear how those elements propagate in the genome, whether autonomous *Mutator* elements drive their mobilization, and how mobilized copies are directed to “TA” microsatellites. An alternative mechanism assuming the propagation of MiMs via homologous recombination, preceded by the formation of extrachromosomal circular DNA (eccDNA) intermediates with microsatellite sequences at MiM ends, was proposed by Franco et al. [56].

### 4.2. Distribution of MITEs in the Carrot Genome

We showed that 16,693 carrot MITEs (54%) reside in genic regions, and 21% of those are co-transcribed with the nearby gene, possibly affecting gene expression or the fate of MITE-containing transcripts. MITEs can interact with genes in multiple ways. Upon insertion in regulatory regions, they may alter gene expression by providing transcription factor binding sites [19]. The presence of polyadenylation signals or splicing sites in the MITE sequence may lead to the formation of new isoforms. On the other hand, MITEs may down-regulate genes by altering methylation through RNA-directed methylation (RdDM) [14,15]. We observed that different carrot MITE groups were enriched in different genic contexts, which may suggest their possible involvement in different regulatory mechanisms. In general, elements inserted into the gene body (UTRs, cds or introns) were often co-transcribed. It was the most apparent in the case of *hAT*-like MITEs, which were inserted into genes relatively more frequently. Here, we demonstrated their engagement in the formation of new isoforms. In the case of two genes, MITE-containing isoforms were prevalent. Such a variability of transcripts may result in a measurable phenotypic effect. For instance, the insertion of a copia retrotransposon into the intron of a tomato gene *Solyc02g079490* produced multiple isoforms without affecting the overall gene expression, but leading to overexpression of non-functional transcripts, ultimately resulting in the accumulation of 2-phenylethanol and giving the tomato fruit a pleasant floral aroma [57]. Carrot *Tourist* elements were preferentially localized in UTRs and regions upstream or downstream from genes, but only insertions residing in UTRs were present in transcripts. In contrast, *Mutator*-like MITEs, enriched not only in intergenic regions but also in introns, were rarely retained in transcripts. *Stowaways* were relatively rarely present in transcripts, despite being the most numerous group of carrot MITEs. However, it should be stressed that the overview of general trends observed for each MITE group was certainly biased by the behavior of the most numerous families. A recent report on carrot *DcStos* indicated strong family-specific preferences for their genomic positioning. Some *DcSto* families were shown to be enriched in genic regions, especially in the vicinity of genes encoding transcription factors [33]. Thus, a detailed characterization of individual families, including information about the expression of nearby genes, is necessary to understand and resolve the involvement of MITEs in the regulation of gene expression.

### 4.3. MITEs in Other Asterid Species

An in silico identification of transposable elements can be biased, and the number of identified repetitive sequences largely depends on the pipeline and stringency of parameters used for analysis. This was why we tested eight Asterid species using the same pipeline we applied for the carrot. The number of families mined from the tomato and potato genomes proved to be consistent with previous reports [26], confirming that the method was adequate, and the carrot genome was indeed characterized by a large diversity and abundance of MITEs. We showed that MITEs were species-specific; however, some MITE families were shared among species from the same botanical family. The number of MITE families shared among species reflected their phylogenetic relationships. We identified 150 carrot MITE families sharing a similarity with MITEs from at least one other Apiaceae species. The intra-family identity in sequence and length decreases over time due to random mutations. The approach we used was based on the 80-80-80 rule for grouping copies into families within the genome, while a less stringent criterion, requiring an identity of at least 60% over 60% of the sequence length, was applied for interspecific comparisons. Detection of nine MITE families shared among Apiaceae, which diverged by about 26 Mya [58], raises a question about their origin. It is possible that they have been retained owing to their functional role in the host genomes. The TE conservation may be related to wiring new transcriptional networks. For example, similar TFBS-carrying MITEs were reported to be shared among species within the *Prunus* and *Solanum* genera [19]. Similarly, *Helitrons* in *A. thaliana* are enriched in PHE1 binding sites, and their insertions up-regulate a number of genes in the endosperm. Some of those *Helitron* insertions are also present in orthologous genes of other Brassicaceae [59]. It was also shown that some ancient TEs identified in Brassicaceae were enriched in gene regulatory networks, e.g., the flowering gene network, suggesting their domestication and conservation for more than 100 million years [60]. Even though the preservation of MITEs in related species is possible, a horizontal transfer cannot be ruled out, as there has been evidence of TE horizontal transfers in animals and plants, and between viruses and their hosts [61,62,63,64].

Our results suggest that some MITEs may play regulatory functions in the carrot and other Apiaceae. We reported a carrot MITE repository and genome annotation providing means for further analysis of MITEs and their effect on the structure and function of carrot genes and genomes. Besides the analysis of the functional role and impact on the genome evolution, insertional polymorphism of MITEs belonging to newly described families can be utilized as molecular markers. Until now, MITE-based molecular markers were successfully used to study carrot diversity and population structure [35,36]. Identification of new MITE families, especially those associated with genes, provides a rich source of transposable element-associated structural variants (TEASVs) that can be used for TE-based genome-wide association mapping (TE-GWAS), an approach recently successfully applied in rice [65].

## 5. Conclusions

Carrot MITEs are exceptionally diverse and abundant. Their localization in the vicinity of genes and presence in transcribed regions points to their possible involvement in the regulation of gene expression and the formation of novel isoforms. The identification and characterization of carrot MITEs provide a basis for further studies on their functional impact. It also facilitates the use of MITE insertion polymorphisms to identify genetic factors associated with important agronomic traits of the carrot.

## Figures and Tables

**Table 1:** MITEs identified in the carrot genome.

MITE	Number of Families	Number of Copies	Mbp	% of Genome	Mean Copy Number per Family	Mean Length of MITE (bp)	Copies in Most Nmerous Family	Genomic Localization						
								Intergenic	Upstream	5′UTR	Cds	Intron	3′UTR	Downstream
*hAT*	144	3637	1.65	0.35%	25	454	217	1506	608	77	31	918	66	431
*Mutator*	128	6308	2.71	0.57%	49	429	542	3019	1148	52	12	1343	58	676
*Stowaway*	56	11,674	2.97	0.63%	208	254	1452	5605	2446	109	11	1991	89	1423
*Tourist*	81	8731	2.78	0.59%	108	319	1353	3867	1803	134	19	1574	110	1224
Unclassified	19	675	0.23	0.05%	36	335	141	335	99	5	2	188	5	41
Total	428	31,025	10.34	2.19%	-	-	-	14,332	6104	377	75	6014	328	3795

**Table 2:** MITEs identified in nine Asterid species.

Plant	Number of MITE Copies	Number of Families (MITE-Hunter)	Occupied Genome Fraction (Mbp)	Fraction of Assembled Genome (%)	Fraction of Genome (%)	Size of Assembly (Mbp)	Genome Size (Mbp)	Reference for the Published Genome Size
celery	52,269	83	27.71	0.83	0.80	3332.58	3470	[47]
pepper	31,405	149	12.43	0.42	0.36	2935.88	3500	[48]
coffee	25,038	184	12.11	1.11	0.93	1094.45	1300	[49]
carrot	31,025	428	10.34	2.45	2.19	421.54	473	[30]
fennel	17,861	186	5.67	0.56	0.42	1010.97	1340	[50]
common sunflower	114,089	342	73.28	2.43	2.09	3010.05	3500	[51]
Java waterdropwort	21,330	217	7.79	0.61	-	1278.51	-	-
tomato	15,150	119	7.07	1.00	0.79	705.93	900	[52]
potato	20,368	251	7.65	0.92	0.91	828.35	844	[53]

## Data Availability

We used the following previously published reference genome assemblies available at NCBI: carrot (GenBank acc. no. GCA_001625215.1), celery (GCA_009905375.1), pepper (GCA_000710875.1), coffee (GCA_003713225.1), fennel (GCA_003724115.1), common sunflower (GCA_002127325.1), Java waterdropwort (GCA_008931105.1), tomato (GCA_000188115.3), potato (GCA_000226075.1), and carrot RNAseq reads from twenty DH1 tissues (PRJNA291977).

## Supplementary Materials

The following are available online at https://www.mdpi.com/article/10.3390/genes12060859/s1, Figure S1: Box-plot showing the number of copies in each group of carrot MITEs. Figure S2: Abundance and genomic localization of carrot MITEs present in transcripts. Figure S3: Relationship between the number of copies in transcribed regions and the total number of copies representing each MITE family. Figure S4: Relative abundance of MITE groups in different genomic regions, divided into present (bolded) or absent in transcripts (*p*-value < 0.001). Figure S5: Number of normalized RNAseq reads (RPM) attributed to each MITE group. Figure S6: Splice variants and differential exon usage for LOC108223373. Figure S7: Splice variants and differential exon usage for LOC108201852. Figure S8: Splice variants and differential exon usage for LOC108227539. Figure S9: Splice variants and differential exon usage for LOC108220652. Figure S10: Splice variants and differential exon usage for LOC108222651. Figure S11: Plot showing the number of MITE copies present in Asterid species (y axis) related to the genome size (x axis). Figure S12: Relationships among MITE families shared by four Apiaceae species. Table S1: Carrot DH1 transcriptomes used for identification of MITEs in transcripts. Table S2: Asterid species used for identification of MITEs. Table S3: Carrot MITEs identified in transcripts. Table S4: Summary of MITEs present in transcripts and inserted in the vicinity/within genes with RPK > 1 and M/g > 1.9. Table S5: List of randomly selected genes containing *hAT*-like MITE insertions assigned to UTRs, cds or introns, tested for MITE-related differential exon usage. Table S6: Number of MITE families shared between Asterid species. Table S7: Number of carrot MITE families shared by two or more Apiaceae species. File S1: A gff3 file showing annotation of mined MITEs in the carrot reference genome. File S2: Characteristics of carrot MITE families, File S3: Detailed results of analysis of isoforms of *hAT*-like MITE containing genes. File S4: List of MITE families shared by two or more Apiaceae species.
