# Supplementary material for: A Global Landscape of Miniature Inverted-Repeat Transposable Elements in the Carrot Genome
Source: Genes (Basel). 2021 Jun 3;12(6):859. doi: 10.3390/genes12060859 (PMC8227079; doi:10.3390/genes12060859)
Supplement: Supplementary file 1 [file genes-12-00859-s001.zip › draft_MITE_fig_tabls_12.05.2021_genes_file_revised.pdf]

Supplementary Table S1. Carrot DH1 transcriptomes used for identification of MITEs in transcripts

No.	SRA Run	Tissue	Name
1	SRR2148979	0.5 mm buds	buds
2	SRR2148980	Bracts (from not opened flower), from 2 cm umbel	bracts_no
3	SRR2148981	Whole flowers (not opened), 2 cm umbel	flowers_no
4	SRR2148982	Bracts (from opened flower), from 2 cm umbel (flower at anthesis)	bracts_o
5	SRR2148983	Whole flowers (opened), 2 cm umbel with flower at anthesis	flowers_o
6	SRR2148984	Leaves stage 1, 0.5-1 cm young sprout	leaves1
7	SRR2148985	Leaves stage 2, 2-2.5 cm leaves, not expanded	leaves2
8	SRR2148986	Leaves stage 3, 7-8 cm leaves, expanded	leaves3
9	SRR2148987	10 cm petiole from leaves stage3	petiole
10	SRR2148988	Hypocotyl from root of 4 cm long, 1.5 cm diameter	hypocotyl
11	SRR2148989	Phloem from root of 4 cm long, 1.5 cm diameter	phloem
12	SRR2148990	Xylem from root of 4 cm long, 1.5 cm diameter	xylem
13	SRR2148991	Fibrous roots	fibrous_roots
14	SRR2148992	Callus	callus
15	SRR2148993	Stressed leaves of 2-2.5 cm at reversible wilting point	leaves_S1
16	SRR2148994	Stressed leaves at reversible wilting point	leaves_S2
17	SRR2148996	Stressed leaves of 7-8 cm at reversible wilting point	leaves_S3
18	SRR2148997	Stressed root, whole storage root	root
19	SRR2148998	Etiolated leaves, yellowish leaves from different stages	leaves_e
20	SRR2148999	Germinating seeds, at the beginning of germination	seeds

Supplementary Table S2. Asterid species used for identification of MITEs

Organism	Family	Assembly	GenBank assembly accession
Apium graveolens (celery)	Apiaceae	ASM990537v1	GCA_009905375.1
Capsicum annuum (pepper)	Solanaceae	Pepper Zunla 1 Ref_v1.0	GCA_000710875.1
Coffea arabica (coffee)	Rubiaceae	Cara_1.0	GCA_003713225.1
Daucus carota (carrot)	Apiaceae	ASM162521v1	GCA_001625215.1
Foeniculum vulgare (fennel)	Apiaceae	FoenVul1.0	GCA_003724115.1
Helianthus annuus (common sunflower)	Asteraceae	HanXRQr1.0	GCA_002127325.1
Oenanthe javanica (Java waterdropwort)	Apiaceae	ASM893110v1	GCA_008931105.1
Solanum lycopersicum (tomato)	Solanaceae	SL3.0	GCA_000188115.3
Solanum tuberosum (potato)	Solanaceae	SolTub_3.0	GCA_000226075.1

Supplementary Table S3 Carrot MITEs identified in transcripts. The percentage of transcribed MITEs over the total copy number assigned to a given genomic location is shown in parentheses.

MITE	Upstream	5'UTR	cds	intron	3'UTR	Downstream	Total
hAT	108 (18%)	40 (52%)	19 (61%)	454 (49%)	47 (71%)	85 (20%)	753 (35%)
Mutator	111 (10%)	16 (31%)	7 (58%)	435 (32%)	25 (43%)	89 (13%)	683 (21%)
Stowaway	210 (9%)	40 (37%)	2 (18%)	536 (27%)	36 (40%)	126 (9%)	950 (16%)
Tourist	156 (9%)	60 (45%)	9 (47%)	591 (38%)	56 (51%)	135 (11%)	1,007 (21%)
UC*	8 (8%)	0 (0%)	0 (0%)	70 (37%)	3 (60%)	1 (2%)	82 (24%)
Total	593 (10 %)	156 (41%)	37 (49%)	2,086 (35%)	167 (51%)	436 (11%)	3,475 (21%)

* unclassified MITEs

Supplementary Table S4. Summary of MITEs present in transcripts and inserted in the vicinity/within genes with RPK>1 and M/g >1.9.

MITE	Upstream	5'UTR	cds	intron	3'UTR	Downstream	Total
hAT	76	34	13	423	41	64	651
Mutator	85	15	6	395	24	69	594
Stowaway	154	35	1	497	36	100	823
Tourist	130	56	7	532	52	109	886
UC*	2	0	0	63	3	0	68
Total	447	140	27	1910	156	342	3022

* unclassified MITEs

Supplementary Table S5. List of randomly selected genes containing *hAT*-like insertions assigned to UTRs, cds or introns, tested for MITE-related differential exon usage.

Gene	MITE name	MITE localization	MITE begining	MITE end	differential exon usage attributed to MITE
LOC108205252	TSD8_10_hAT_2	5'UTR	2582589	2582974	no
LOC108205040	TSD8_11_hAT_11	intron	5859963	5860820	no
LOC108204960	TSD8_4_hAT_3	intron	12557668	12558094	no
LOC108201852	TSD8_4_hAT_3	3'UTR	35579083	35579531	yes
LOC108193394	TSD8_4_hAT_2	intron	36992336	36992767	no
LOC108204645	TSD8_TA_hAT_3	Exon/3'UTR	44399269	44399644	no
LOC108204661	TSD8_TA_hAT_4	3'UTR	49351554	49352292	no
LOC108209102	TSD8_TA_hAT_1	3'UTR	4525264	4526003	no
LOC108206945	TSD8_4_hAT_4	5'UTR	18725016	18725391	no
LOC108208893	TSD8_9_hAT_1	Exon/3'UTR	27257411	27257847	no
LOC108205995	TSD8_4_hAT_3	intron	36381678	36382125	no
LOC108210606	TSD8_3_hAT_1	intron	16334378	16334560	yes
LOC108215111	TSD8_13_hAT_3	intron	47645112	47645858	no
LOC108214355	TSD8_3_hAT_1	5'UTR	47844534	47844695	no
LOC108216049	TSD8_TA_hAT_38_4	5'UTR	20748194	20749033	no
LOC108219293	TSD8_9_hAT_1	5'UTR	30009753	30010194	no
LOC108219489	TSD8_TA_hAT_2	5'UTR	31470172	31470436	no
LOC108223373	TSD8_TA_hAT_23	3'UTR	278545	279122	yes
LOC108223352	TSD8_TA_hAT_18	5'UTR	4295467	4295992	no
LOC108222651	TSD8_4_hAT_3	Exon/3'UTR	12305668	12306116	yes
LOC108223859	TSD8_4_hAT_3	intron	14435656	14436103	no
LOC108220048	TSD8_5_hAT	intron	24364637	24365081	no
LOC108224034	TSD8_3_hAT_3	3'UTR	31196333	31196631	no
LOC108222921	TSD8_10_hAT_11	intron	32474494	32474807	no
LOC108220652	TSD8_13_hAT_1	5'UTR	39521923	39522573	no
LOC108227956	TSD8_4_hAT_2	intron	20667783	20668222	no
LOC108227343	TSD8_TA_hAT_5	3'UTR	22362164	22362493	no
LOC108227593	TSD8_4_hAT_2	intron	23903383	23903821	no

LOC108227932	TSD8_TA_hAT_34_8	5'UTR	28774483	28775008	no
LOC108224525	TSD8_4_hAT_7	intron	35521777	35522278	no
LOC108225178	TSD8_4_hAT_1	5'UTR	36006616	36006802	no
LOC108227539	TSD8_4_hAT_3	intron/exon	36524270	36524718	yes
LOC108193484	TSD8_TA_hAT_17	5'UTR	5236057	5236475	no
LOC108193832	TSD8_TA_hAT_5	5'UTR	28108440	28108722	no
LOC108199912	TSD8_TA_hAT_28	3'UTR	23374509	23375160	no
LOC108202676	TSD8_7_hAT	intron	2167934	2168547	no
LOC108202676	TSD8_7_hAT	intron	2169299	2169914	no
LOC108201955	TSD8_16_hAT_2	intron/exon	5439962	5440558	no
LOC108201989	TSD8_TA_hAT_37_2	intron	8896662	8897383	no
LOC108203014	TSD8_4_hAT_1	3'UTR	9134444	9134625	no

Supplementary Table S6. Number of MITE families shared between Asterid species. To identify similar MITEs, the overlap between sequence depending on the length of the blast hit was one criterion, therefore results are presented for both directions.

blast query	blast hit								
	celery	pepper	coffee	carrot	fennel	sunflower	Java waterdropwort	tomato	potato
celery	-	-	-	36	40	-	22	-	-
pepper	-	-	-	-	-	-	-	10	16
coffee	-	-	-	-	-	-	-	-	-
carrot	33	-	-	-	84	-	50	-	-
fennel	37	-	-	84	-	-	35	-	-
sunflower	-	-	-	-	-	-	-	-	-
Java waterdropwort	18	-	-	51	30	-	-		
tomato	-	12	-	-	-	-	-	-	122
potato	-	18	-	-	-	-	-	107	-

Supplementary Table S7. Number of carrot MITE families shared with *Apiaceae* species.

Plant species	*hAT*	*Mutator*	*Stowaway*	*Tourist*	UC	Total
carrot, fennel, celery, Java waterdropwort	2	0	8	2	0	9
carrot, Java waterdropwort, celery	0	0	3	2	0	5
carrot, Java waterdropwort, fennel	1	2	9	7	1	20
carrot, celery, fennel	0	0	13	3	2	18
carrot, fennel	12	5	17	14	5	53
carrot, Java waterdropwort	2	4	11	9	1	27
carrot, celery	0	3	4	4	4	15
Total	17	14	63	40	13	147

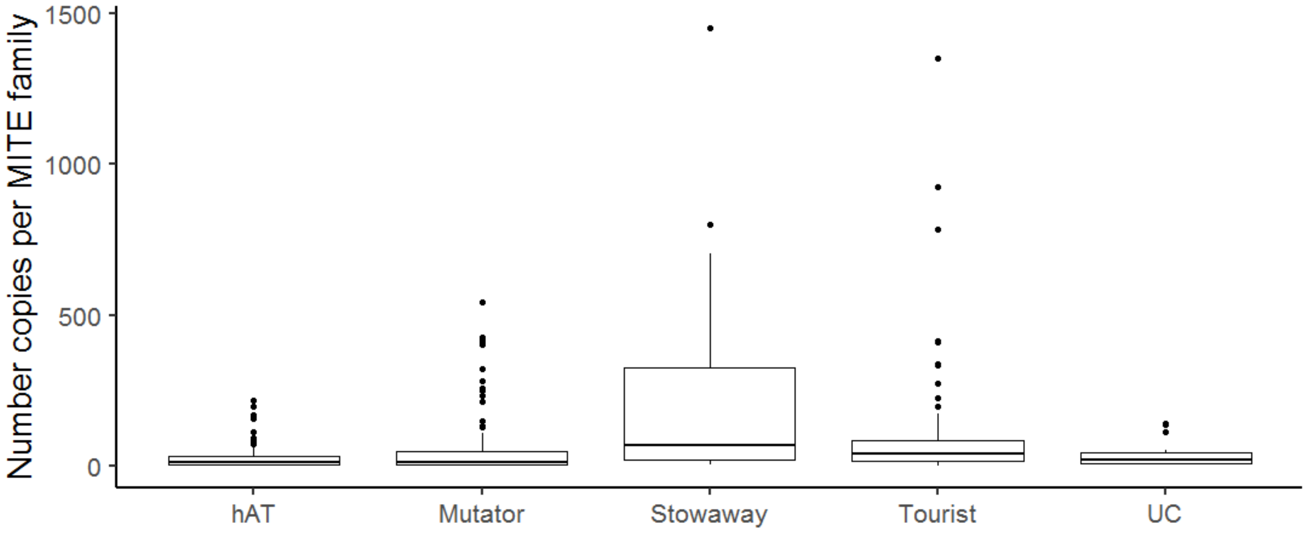

Supplementary Figure S1. Box-plot showing the number of copies in each group of carrot MITEs. UC stands for unclassified MITEs.

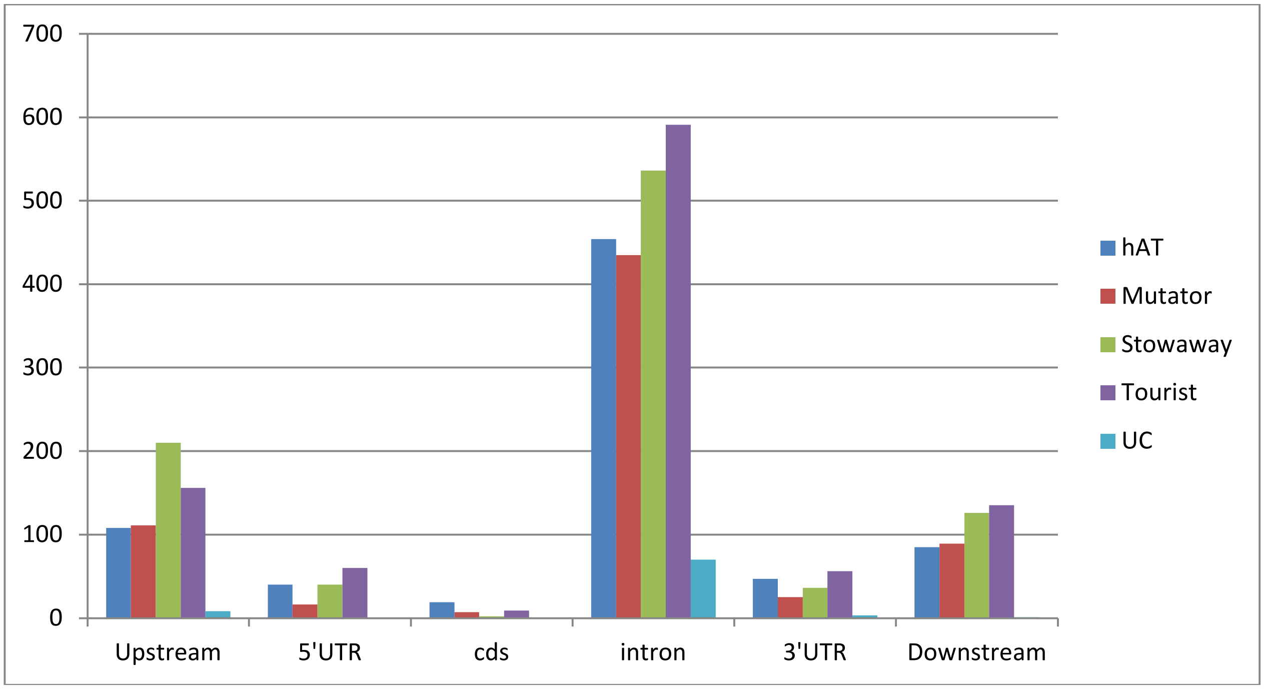

Supplementary Figure S2. Abundance and genomic localization of carrot MITEs present in transcripts. UC stands for unclassified MITEs.

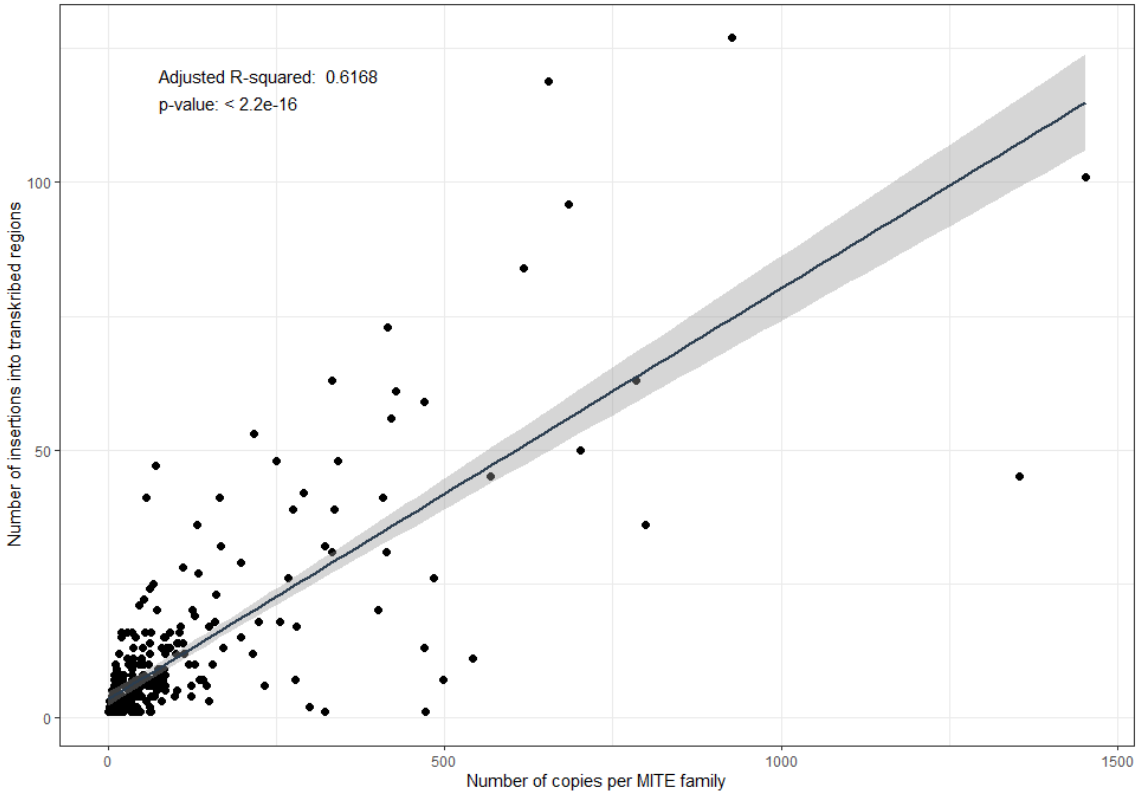

Supplementary Figure S3. Relationship between the number of copies in transcribed regions and the total number of copies representing each MITE family.

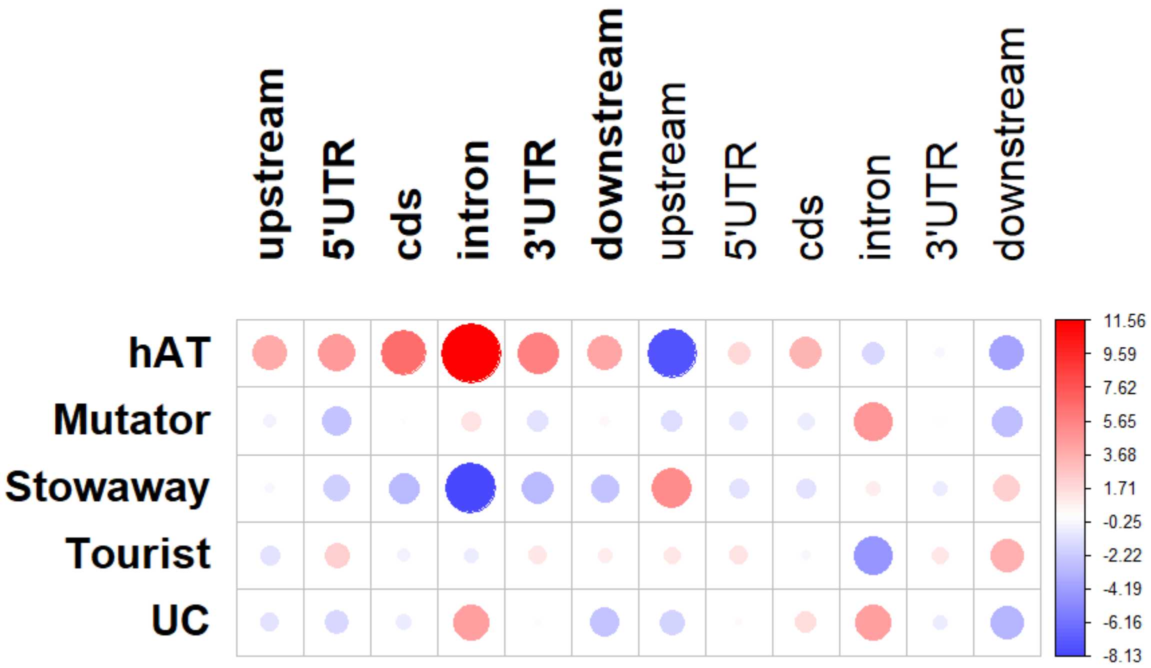

Supplementary Figure S4. Relative abundance of MITE groups in different genomic regions, divided into present (bolded) or absent in transcripts (p-value <0.001). Color scale reflects deviations from the average value. Circle size is proportional to the contribution of each test to the total Pearson chi-squared score. UC stands for unclassified MITEs.

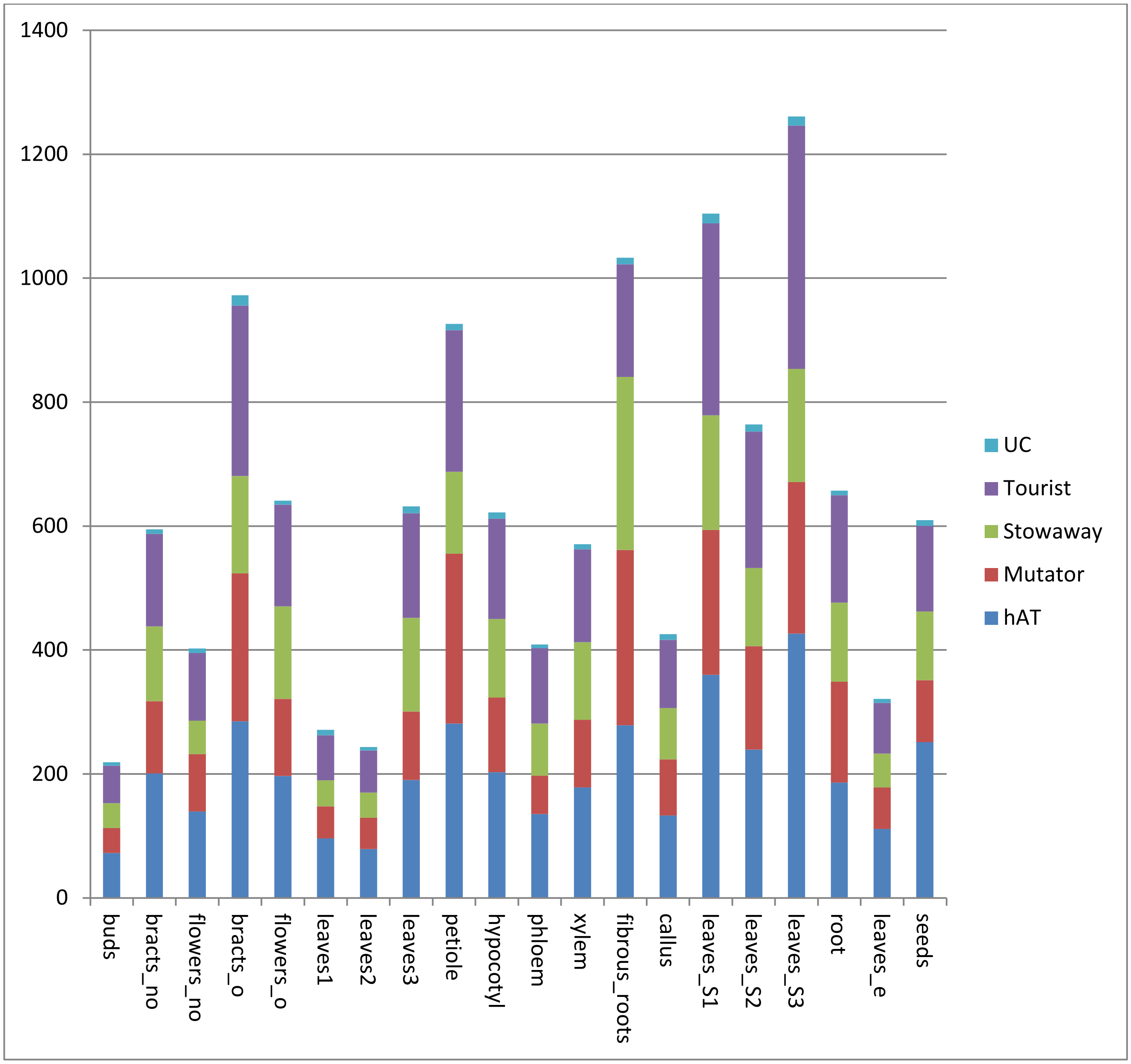

Supplementary Figure S5. Number of normalized RNAseq reads (RPM) attributed to each MITE group. Detailed description of RNA samples is provided in the Supplementary table 2. UC stands for unclassified MITEs.

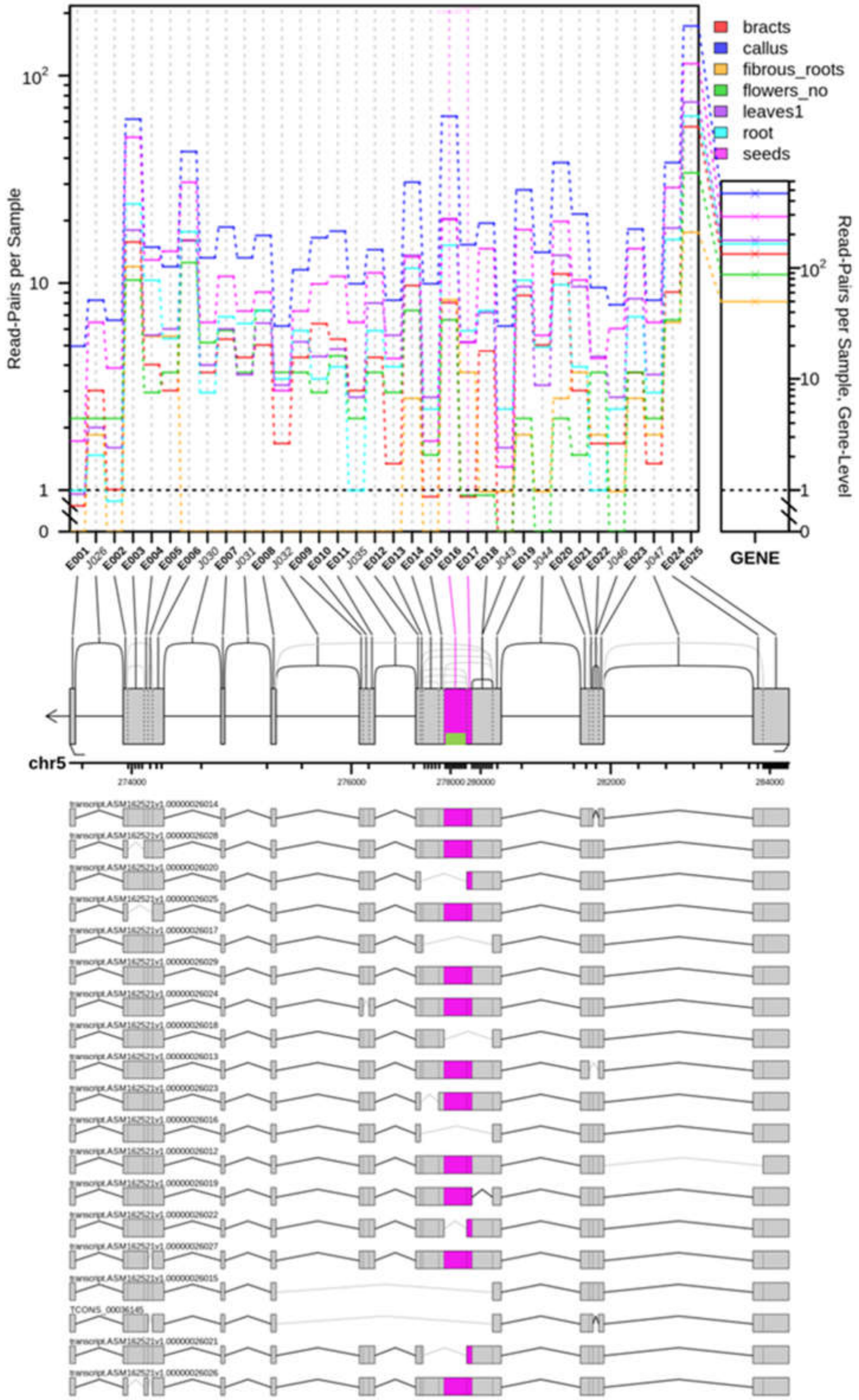

Supplementary Figure S6. Splice variants and differential exon usage for LOC108223373. Differentially spliced exons are colored in purple and the position of a *hAT*-like element is highlighted in green. Novel isoforms are labeled as TCONS_XXX. Detailed results/descriptions are provided in the Supplementary file 3.

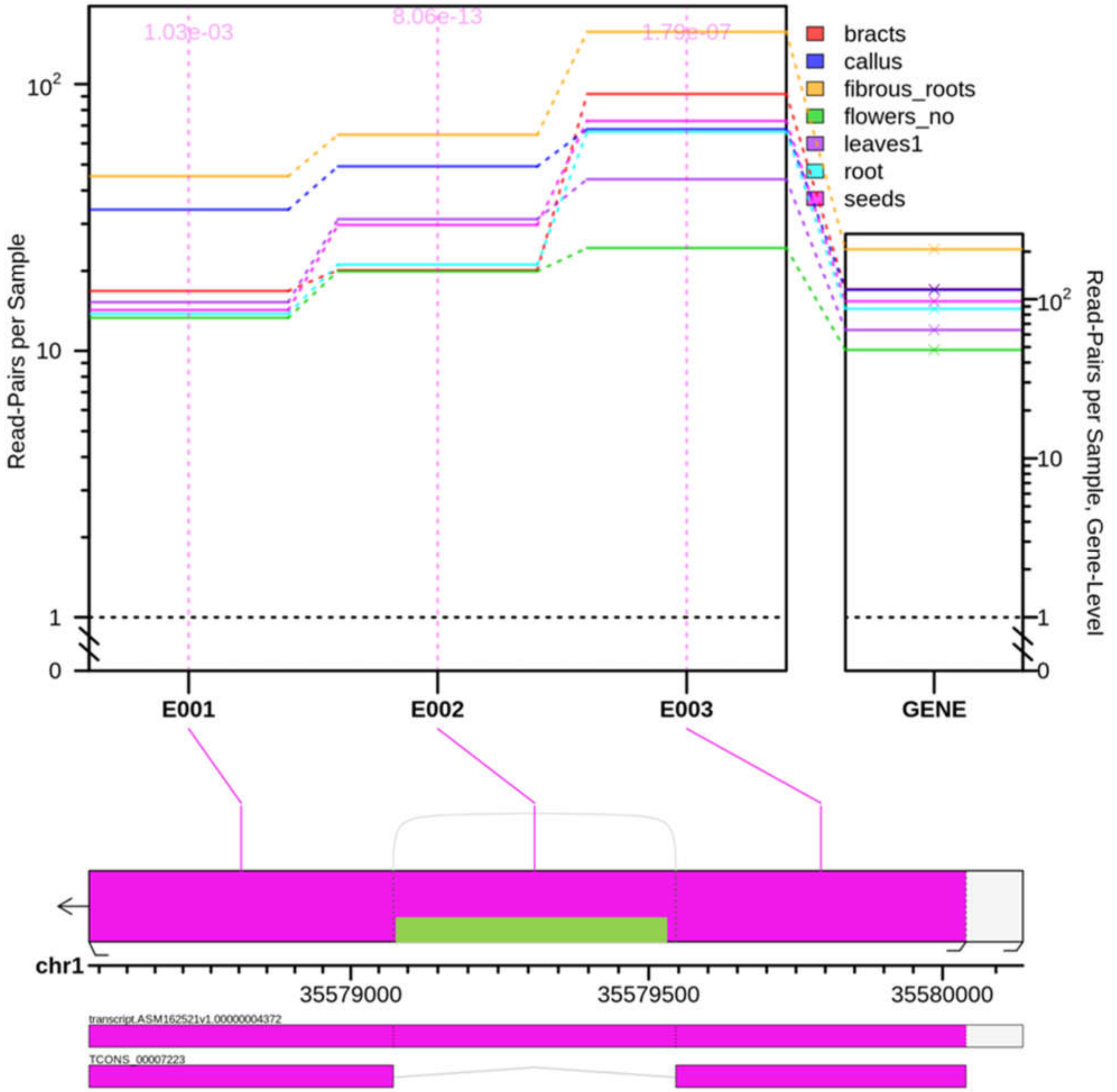

Supplementary Figure S7. Splice variants and differential exon usage for LOC108201852. Differentially spliced exons are colored in purple and the position of a *hAT*-like element is highlighted in green. Novel isoforms are labeled as TCONS_XXX. Detailed results/descriptions are provided in the Supplementary file 3.

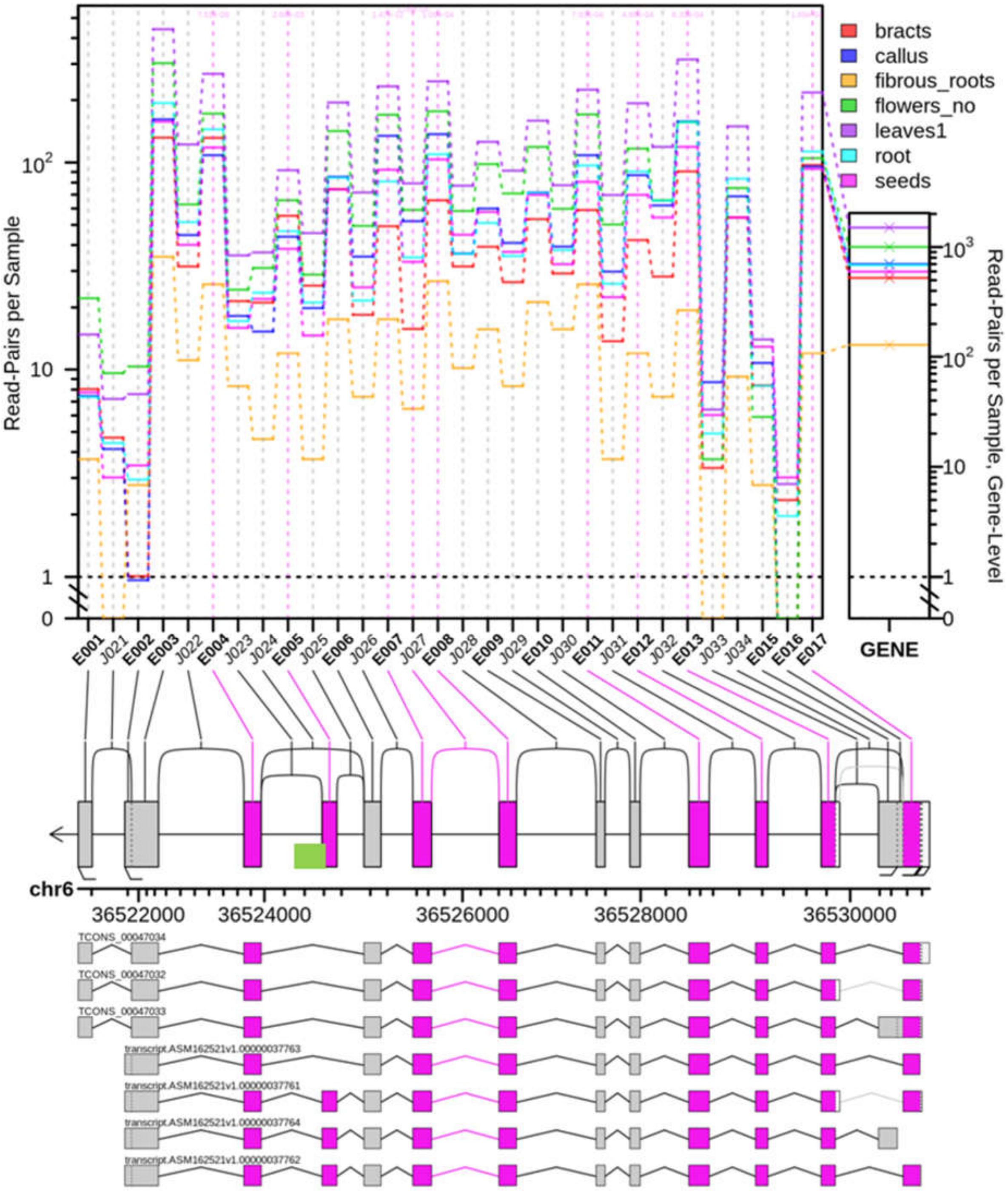

Supplementary Figure S8. Splice variants and differential exon usage for LOC108227539. Differentially spliced exons are colored in purple and the position of a *hAT*-like element is highlighted in green. Novel isoforms are

labeled as TCONS_XXX. Detailed results/descriptions are provided in the Supplementary file 3.

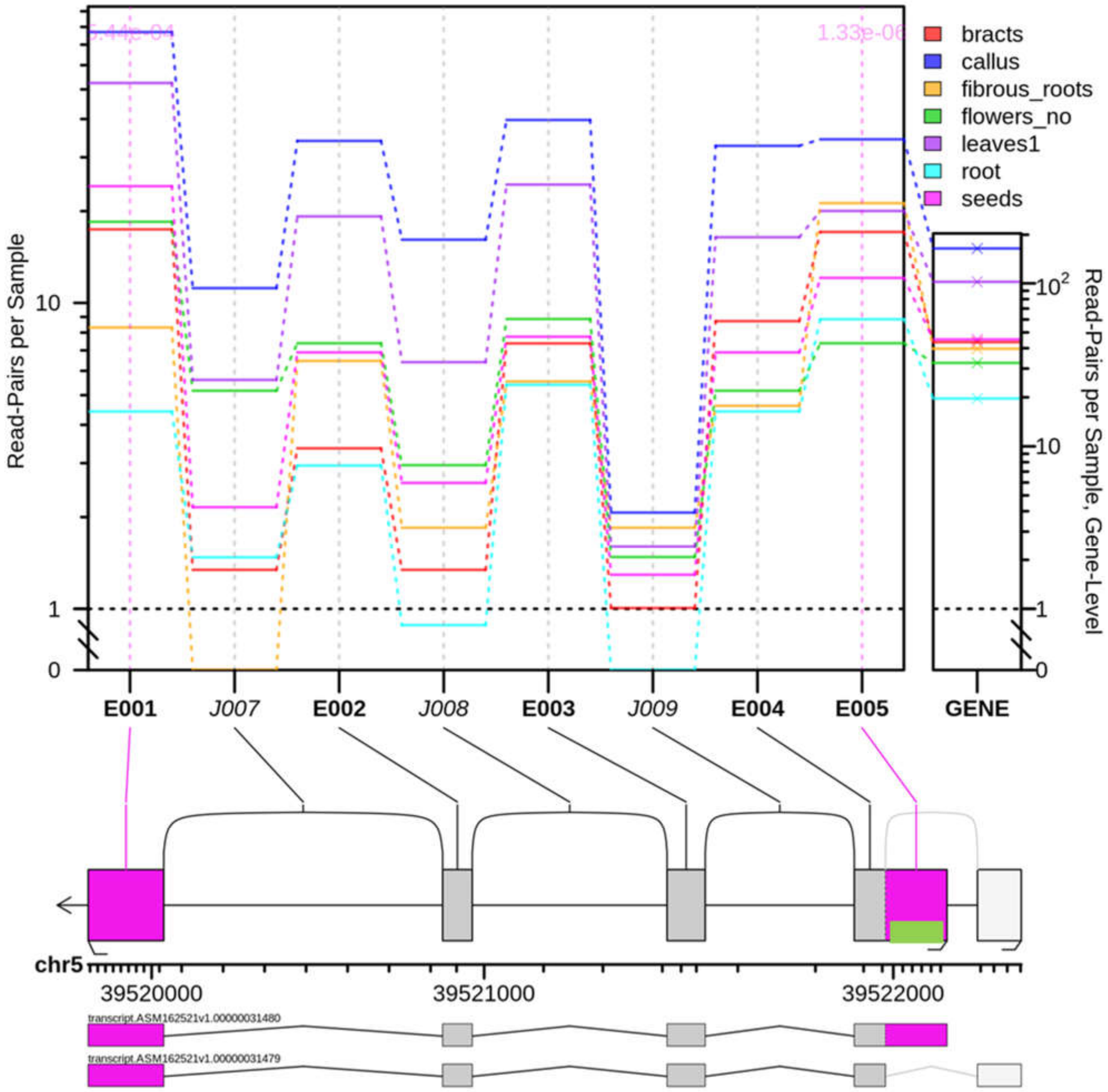

Supplementary Figure S9. Splice variants and differential exon usage for LOC108220652. Differentially spliced exons are colored in purple and the position of a *hAT*-like element is highlighted in green. Novel isoforms are labeled as TCONS_XXX. Detailed results/descriptions are provided in the Supplementary file 3.

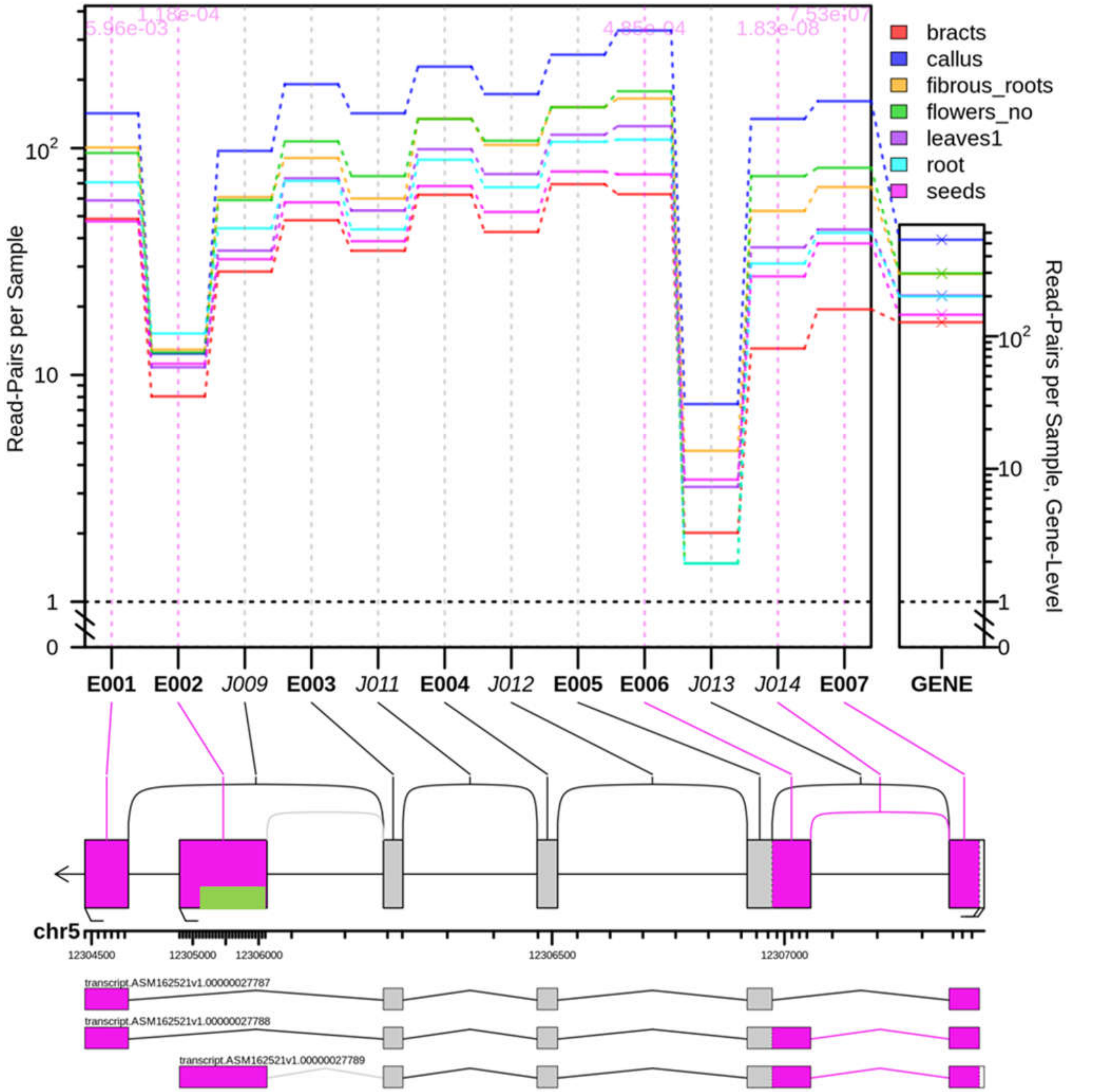

Supplementary Figure S10. Splice variants and differential exon usage for LOC108222651. Differentially spliced exons are colored in purple and the position of a *hAT*-like element is highlighted in green. Novel isoforms are labeled as TCONS_XXX. Detailed results/descriptions are provided in the Supplementary file 3.

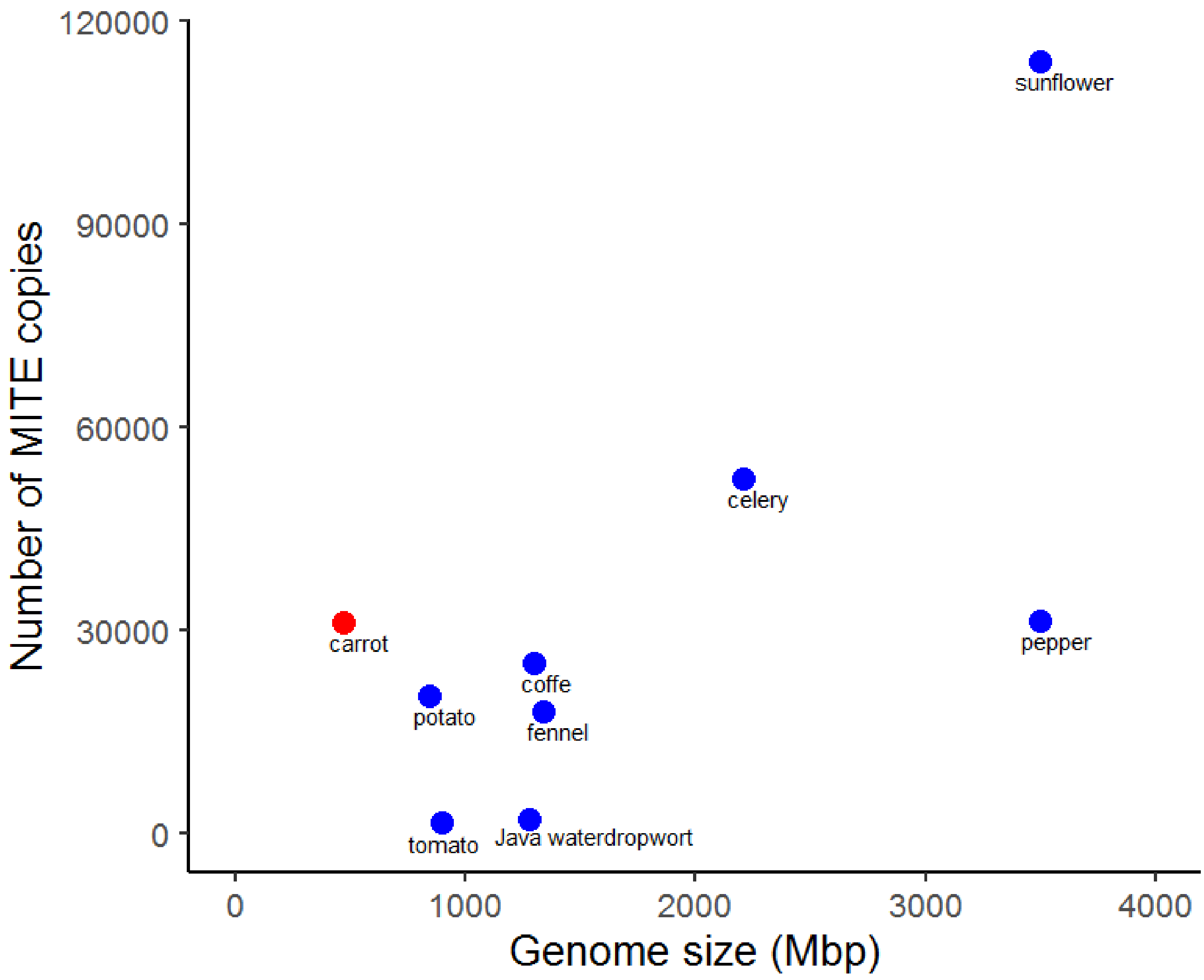

Supplementary Figure S11. Plot showing the number of MITE copies present in *Asterid* species (y axis) related to the genome size (x axis).

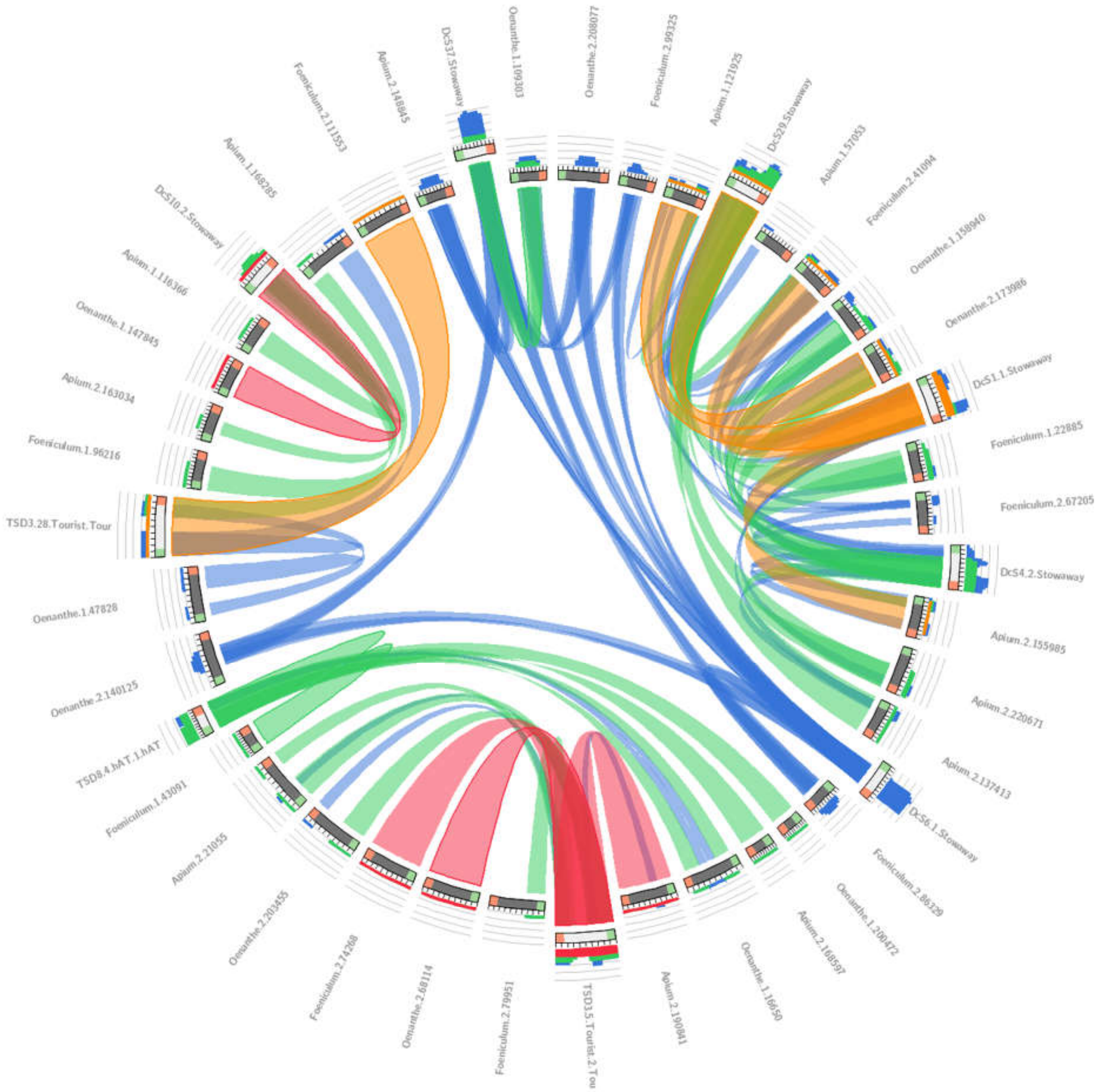

Supplementary Figure S12. Relationships among MITE families shared by four Apiaceae species. Ribbon colors represent 'score/max' ratio coloring with blue<=0.25, green<=0.50, orange<=0.75, red>0.75.
